# Uncoupling Protein 2 Regulates Palmitic Acid-Induced Hepatoma Cell Autophagy

**DOI:** 10.1155/2014/810401

**Published:** 2014-08-04

**Authors:** Jiaxin Lou, Yunjiao Wang, Xuejiang Wang, Ying Jiang

**Affiliations:** Department of Pathophysiology, Capital Medical University, 10 You An Men Wai Xi Tou Tiao, Beijing 100069, China

## Abstract

Mitochondrial uncoupling protein 2 (UCP2) is suggested to have a role in the development of nonalcoholic steatohepatitis (NASH). However, the mechanism remains unclear. Autophagy is an important mediator of many pathological responses. This study aims to investigate the relationship between UCP2 and hepatoma cells autophagy in palmitic acid- (PA-) induced lipotoxicity. H4IIE cells were treated with palmitic acid (PA), and cell autophagy and apoptosis were examined. UCP2 expression, in association with LC3-II and caspase-3, which are indicators of cell autophagy and apoptosis, respectively,was measured. Results demonstrated that UCP2 was associated with autophagy during PA-induced hepatic carcinoma cells injury. Tests on reactive oxygen species (ROS) showed that UCP2 overexpression strongly decreases PA-induced ROS production and apoptosis. Conversely, UCP2 inhibition by genipin or UCP2 mRNA silencing enhances PA-induced ROS production and apoptosis. Autophagy partially participates in this progress. Moreover, UCP2 was associated with ATP synthesis during PA-induced autophagy. In conclusion, increasing UCP2 expression in hepatoma cells may contribute to cell autophagy and antiapoptotic as result of fatty acid injury. Our results may bring new insights for potential NASH therapies.

## 1. Introduction

The uncoupling proteins (UCPs) belong to the mitochondrial anion transporter superfamily in the inner mitochondrial membrane [1, 2], and UCP2 is believed to play a role in adaptive thermogenesis and lipid metabolism [3]. Moreover, the promoter region of UCP2 contains Sp1, double E-box, and sterol response elements, which could explain why UCP2 is upregulated in response to high levels of fatty acids, obesity, fasting, leptin, and other conditions [4, 5]. Although normal healthy hepatocytes do not express UCP2, there is an increase in expression of this protein following oxidative stress and steatosis [6]. It has been suggested that UCP2 has a role in the development of nonalcoholic fatty liver disease (NAFLD). However, the mechanism remains unclear. UCP2 has been reported to play a role in antioxidant defense [7], as inhibition with the specific inhibitor genipin or inhibition by UCP2 siRNA increases mitochondrial ROS levels, while overexpression of UCP2 diminishes ROS production [8]. These findings suggest that UCP2 might influence the development of NAFLD by regulating ROS production.

Nonalcoholic fatty liver disease (NAFLD) is the most common form of chronic fatty liver disease in Western countries. NAFLD refers to a wide spectrum of liver damage from steatosis to nonalcoholic steatohepatitis (NASH) and, lastly, cirrhosis. NASH is known to be a significant cause of cryptogenic cirrhosis, and therefore it has attracted more attention in the past few years. Studies have shown that FFAs-induced lipotoxicity has been documented to play an essential role in the pathogenesis of NASH [9], and our latest study has shown that palmitic acid- (PA-) induced apoptosis plays an important role in the pathogenesis and development of NASH [10]. At present there is accumulating evidence suggesting that autophagy is involved in the physiological and pathological responses of cells to lipid stimulation [4, 5]. With the evidence that autophagy could regulate hepatic lipid stores [11], and with loss of autophagic function, white adipocyte differentiation was blocked* in vitro* and white adipose tissue mass was markedly decreased* in vivo*. Decreased lipid storage occurred with these changes, and this effect along with an increase in the mass of normal brown adipose tissue led to a significantly increased rate of fatty acid *β*-oxidation [12]. A previous study has shown that PA also triggers autophagy responses following hepatic lipotoxicity [13], which implies that autophagy may be involved in the etiology of NASH.

Autophagy is a lysosome-mediated degradation process for nonessential or damaged cellular constituents. It has a multistep process, including the formation of double-membrane vesicles known as autophagosomes [14]. Moreover, autophagy has been shown to have a critical role in the regulation of hepatocellular lipid accumulation and liver injury during oxidative stress [15].* In vivo* studies have indicated that starvation-stimulated macroautophagy provides the liver with a lipid challenge in the form of increased serum FFAs and that the autophagic pathway selectively targets lipids for breakdown in response to this physiological stimulus [11]. Moreover, UCP2 has been shown to trigger ROS-dependent autophagic cell death in pancreatic adenocarcinoma cells [16]. These studies prove that UCP2 probably mediates FFA-induced hepatocyte autophagy.

Although the mechanism of how did UCP2 mediate PA-induced autophagy in hepatocytes remains unclear, insights into these mechanisms may be useful in designing effective management strategies in dealing with NASH. Therefore, in the present study, we tested the autophagic responses and the underlying mechanisms following PA-induced injury. We observed the induction of autophagy by PA and UCP2 overexpression decreased ROS production. Moreover, UCP2 could enhance hepatoma autophagy, such that UCP2-mediated autophagy induced by PA was first found to serve as an antiapoptotic mechanism to oppose the lipotoxic effects. These results indicate that modulation of UCP2 can serve as a potential experimental therapy for NASH caused by the lipotoxic effects of PA.

## 2. Materials and Methods

### 2.1. Cell Culture and PA Treatment

H4IIE cells, a rat hepatoma cell line, were cultured in Dulbecco's modified Eagle's medium (DMEM; Invitrogen) with 10% (v/v) fetal bovine serum (Hyclone, Rockford, IL, USA), penicillin (100 UI/mL), and streptomycin (100 UI/mL). All cell cultures were maintained in a 37°C incubator with 5% (v/v) CO_2_. To induce cellular damage, 250 *μ*M PA (Sigma, St. Louis, MO, USA) was added to serum-free DMEM medium after the cells grew to ~70–80% confluence. PA-BSA (bovine serum albumin) conjugate was prepared as described previously [17]. In brief, a 100 mM solution of PA in 0.1 M NaOH was incubated at 80°C, and fatty acid soaps were then complexed with 10% (w/v) BSA in phosphate buffered saline (PBS) at a 3.5 : 1 molar ratio of PA to fatty acid free BSA (Wako, Japan). The BSA was used as a vehicle control. CQ (Sigma, USA) was used to block lysosomal function, and the later degradation stage of autophagy was used to measure autophagic flux in cells.

### 2.2. Electron Microscopy

H4IIE cells were seeded on 100 mm plates at a density of 10^6^ cells/plate. After the respective treatments for 6 h, cells were fixed with 3% (v/v) glutaraldehyde and washed three times with 0.1 M phosphate buffer (pH 7.4). Cells were postfixed with 1% (v/v) osmic acid followed by dehydration with an ascending series of alcohol before being embedded in araldite for 24 h. After dehydration, thin sections were cut and stained with uranyl acetate and lead citrate. Digital images were obtained using a JEM 1016CX electron microscope. Random images were obtained.

### 2.3. Cell Proliferation Assay

**Figure 1:**
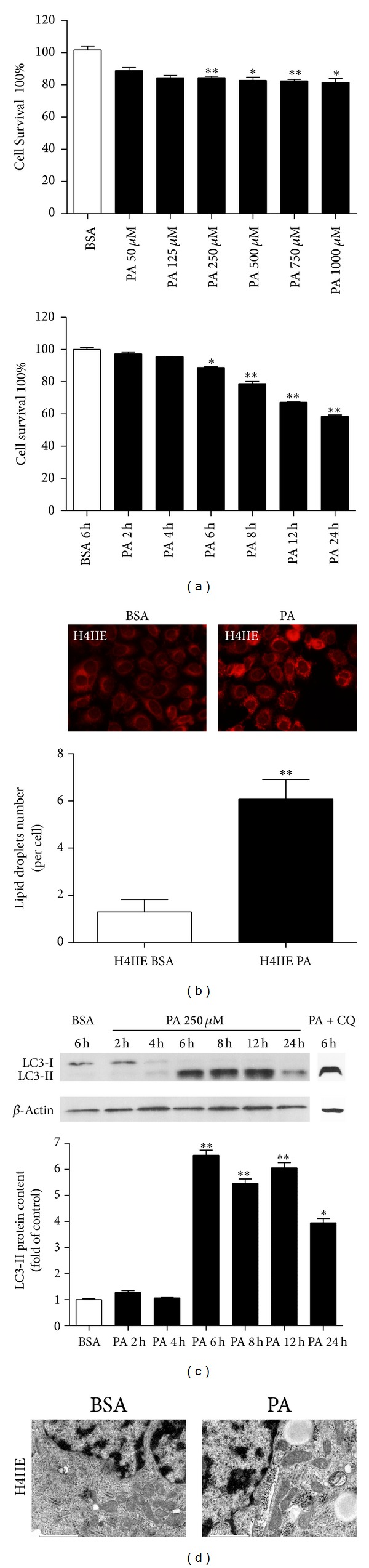
PA induces autophagy in H4IIE cells. (a) H4IIE cells were treated with PA (6 h) conjugated to fatty acid-free BSA at different concentrations, or H4IIE cells were treated with PA (250 *μ*M) conjugated to fatty acid-free BSA at different time points. H4IIE cells treated with BSA acted as a control. After treatments, cells were stained and subjected to the WST-1 assay. (b) Intracellular lipid accumulation was assessed with Nile red staining. PA-induced autophagy (250 *μ*M) of H4IIE cells exhibited numerous small discrete bodies distributed throughout the cytoplasm (objective lens, ×40). (c) H4IIE cells were treated with PA (250 *μ*M) conjugated to fatty acid-free BSA for 2, 4, 6, 8, 12, and 24 h as indicated. Cells treated with BSA acted as a control. After the treatment, cell lysates were collected and subjected to western blotting. (d) H4IIE cells were treated with BSA, PA (250 *μ*M), or PA + CQ (10 *μ*M) for 6 hours before being processed; then electron microscope was performed at 40,000x magnification. All values are the means ± SD of three independent experiments each performed in triplicate.

Cells were seeded in 96-well plates (10^5^ cells/well). After 24 h, cells were treated with various compounds and further incubated for the indicated times (see legends to Figure 1(a)). At the end of the treatments, cells were stained with the cell proliferation reagent WST-1 (Roche, Germany). The dye was solubilized in serum-free DMEM medium and measured photometrically at 450 nm to determine cell growth. Three independent experiments were performed for each assay condition.Cell proliferation was measured using a WST-1 kit according to manufacturer's instruction (Roche, Germany).

### 2.4. Analysis of Intracellular Reactive Oxygen Species

Intracellular reactive oxygen species (ROS) generation was measured with diacetylated 2′,7′-dichlorofluorescein (DCFH-DA, Nanjing Jiancheng Bioengineering Institute, China). In brief, 10^6^ cells were incubated in 60 mm plates and, 24 h later, treated with various compounds, as indicated in the legends to figures. Cells were incubated with 10 *μ*M of DCFH-DA for 20 min at 37°C, and the DCF fluorescence was measured by flow cytometry (Becton Dickinson FACScan, USA) as previously described in detail [18]. Data were analyzed using Cellquest software (Becton Dickinson). All data are presented as the mean of three independent experiments.

### 2.5. Hepatic ATP Level

Cells were seeded in 96-well plates (10^5^  cells/well). After 24 h, cells were treated as indicated. At the end of the treatments, protein concentrations of the lysates were determined using a bicinchoninic acid protein assay kit (Pierce, Rockford, IL, USA), and ATP content was measured using a CellTiter-Glo2.0 assay kit (Promega, USA).

### 2.6. Real-Time PCR

Total RNA from cells was extracted using TRIZOL (Invitrogen, Carlsbad, CA, USA). The first strand of cDNA was synthesized from 5 *μ*g RNA (Superscript III cDNA Synthesis Kit, Invitrogen). The mRNA for UCP2 and 18S was estimated by quantitative real-time PCR using a SYBR Green PCR Kit (Roche). Quantitative RT-PCR was performed with a Prism 7300 Sequence Detecting System (Applied Biosystems). UCP2 primer sequences were as follows: forward primer 5′-AGCAGTTCTACACCAAGGGC-3′, reverse primer 5′-TGGAAGCGGACCTTTACCAC-3′and 18s primer sequences were as follows: forward primer 5′-GTAACCCGTTGAACCCCATT-3′, reverse primer 5′-CCATCCAATCGGTAGTAGCG-3′.

### 2.7. Immunoblot Analysis

Cells were washed in PBS, and whole-cell extracts were prepared in lysis buffer (Tris-HCl (20 mM), pH 7.4, NaCl (150 mM), and glycerol (10% (v/v)), Nonidet P-40 (0.2%), EGTA (1 mM), EDTA (1 mM), PMSF (1 mM), NaF (10 mM), leupeptin (20 mM), aprotinin (5 mg/mL), and sodium orthovanadate (1 mM)) and centrifuged at 8,000 ×g for 15 min. Protein concentrations were measured using the BCA assay (Pierce). Protein (50 *μ*g) was separated on a 15% (w/v) sodium dodecyl sulphate polyacrylamide gel and then transferred to PVDF membranes (Millipore, Billerica, MA, USA). Membranes were incubated overnight with primary antibodies specific to UCP2 (1 : 1000, Biolegend), p-AMPK (1 : 2000, Cell Signaling Technology), caspase-3 (1 : 1000, Cell Signaling Technology, Beverly MA, USA), and LC3 (1 : 2000, Cell Signaling Technology) at 4°C overnight. The positive reaction against these antibodies was visualized by enhanced chemiluminescence (ECL, Santa Cruz) reagent, followed by exposure to Kodak X-Omat X-ray film. After rinsing the membranes with acetonitrile for 10 min, the membranes were rehybridized with antibodies against *β*-actin (1 : 2000, Cell Signaling Technology) as the loading control. Relative density of protein bands was determined using ImageJ software (National Institutes of Health, USA).

### 2.8. Overexpression and Silencing of UCP2

UCP2 overexpression experiments were performed using a pcDNA3.1+ expression vector containing the rat cDNA of UCP2 that we designed previously. The cells were transfected with 5 *μ*g of either the UCP2-bearing vector or control vector with Lipofectamine 2000 (Roche Diagnostic) following the manufacturer's recommendation. Eight hours after transfection, cells were selected using G418 sulphate (600 *μ*g/mL) for 21 days. The cell colonies resistant to G418 were harvested. Stably expressing UCP2-transfected cells were cultured for further studies. UCP2 silencing experiments were carried out with specific small interfering (si) (5′-GUGGUCAAGACGAGAUAUATTUAUAUCUGUCUUGACCACTT-3′) RNA targeting UCP2 mRNA and a nontargeting (NT) siRNA (5′-UUCUCCGAACGUGUCACGUTTACGUGACACGUUCGGAGAATT-3′) purchased from Invitrogen Technologies (Shang Hai, China). Cells were transiently transfected with siRNA according to the manufacturer's protocol (Invitrogen Technologies).

### 2.9. Fluorescence Microscopy

For fluorescence microscopy, cells were cultured in 24-well plates with microscope cover glass. After the designated treatments, cells were fixed with 3% (w/v) paraformaldehyde in PBS. For quantification of autophagic cells, LC3 punctate dots were determined from triplicates by counting at least 60 cells. DAPI (1 *μ*g/mL) was used to detect fragmented and condensed nuclei. Images were acquired with a laser scanning confocal microscope (LEICA TCS SP5). Intracellular lipid droplets were stained as previously described [10]. In brief, cells were stained with Nile red at room temperature; then lipid droplets were obtained using an inverted Olympus fluorescence microscope.

### 2.10. Apoptotic Analysis

Cells were plated in 60 mm plates. After attachment, cells were incubated with 250 *μ*M of PA for 24 h. Cell apoptosis was analyzed using the Annexin V-FITC/PI Apoptosis Kit (Biosea, Beijing, China) according to the instructions and measured by flow cytometry. Data are presented as the mean of three determinations. Cells were seeded in 24-well plates. After being treated as indicated, cells were fixed using 4% paraformaldehyde, and the TUNEL (Promega, USA) manufacturer's protocol was followed. Cells were observed under confocal microscopy and then averaged by the number of TUNEL positive cells/100 cells.

### 2.11. Statistical Analysis

Data are presented as the mean ± SD. Analyses were performed using SPSS 13.0 software, and graphs were performed using Prism 5 software. *P* values < 0.05 or 0.01 are indicated as (∗) or (∗∗), respectively.

## 3. Results

### 3.1. PA Induces Autophagy Activation

To evaluate the effects of PA on intracellular autophagy in H4IIE cells, we first performed WST-1 assays on H4IIE cells treated with PA at different concentrations and different times. Figure 1(a) shows that treatment with PA resulted in a decrease in the levels of cell growth for up to 24 h when compared to control cells treated with fatty acid-free BSA (Wako, Japan). As shown in Figure 1(b), cells were stained by Nile red. We observed an increased number of intracellular lipid droplets in H4IIE cells treated with PA compared to BSA. Western blotting revealed a significant increase in the levels of LC3-II for up to 24 h in comparison to control cells. It is indicated that that PA could increase the levels of LC3-II upon a time course (Figure 1(c)). After inhibition of the late phase of autophagic process by CQ, PA treatment also increased the LC3-II level. However, western blotting revealed that treatment with OA could increase the levels of LC3-II upon a time course (see Supplementary SFigure 4  in Supplementary Material available online at http://dx.doi.org/10.1155/2014/810401), but far more less when compared with the PA-treated cells.

To further confirm the observation that PA treatment does indeed induce autophagy in H4IIE cells, transmission electron microscopy studies were performed under PA treatment (Figure 1(d)). Autophagosome-like vacuoles were hardly seen in BSA-treated control cells. In contrast, we observed an increase in the formation of autophagosome-like structures and lysosomes in PA-treated cells.

### 3.2. UCP2 Partially Mediated PA-Induced Autophagy

**Figure 2:**
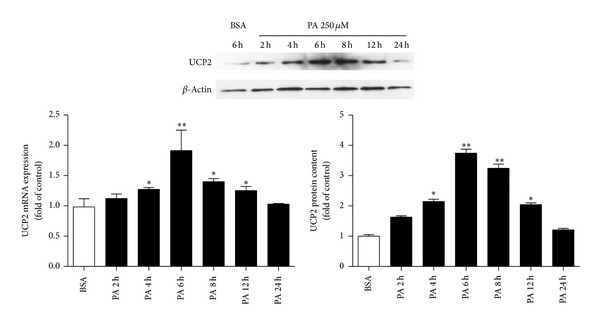
The effects of PA on UCP2 expression. H4IIE cells were treated with PA (250 *μ*M) conjugated to fatty acid-free BSA for 2, 4, 6, 8, 12, and 24 h as indicated. The mRNA level of UCP2 was normalized to 18 s. This ratio was set as 100% with respect to the BSA control. The protein level of UCP2 was normalized to *β*-actin, and this ratio was set as 100% with respect to the BSA control. Data are expressed as the mean ± SD of three independent experiments each performed in triplicate.

To evaluate if PA treatment could also impact UCP2, we monitored UCP2 mRNA and protein expression by real-time PCR and western blotting. Results revealed that the UCP2 mRNA and protein levels were both increased in H4IIE cells treated with 250 *μ*M PA for 6 h compared to the vehicle control (Figure 2), suggesting that the upregulation of UCP2 expression is mediated by PA in hepatoma cells.

**Figure 3:**
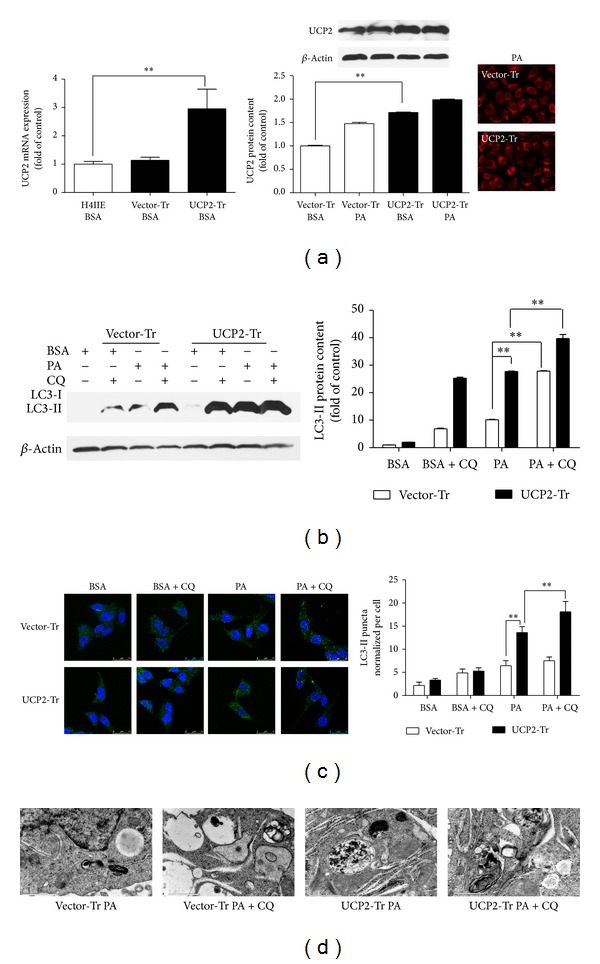
UCP2 overexpression enhanced PA-mediated autophagy. H4IIE cells were transfected with UCP2-bearing plasmid and control vector plasmid and then treated with 250 *μ*M PA for 6** **h, with or without CQ (10**** *μ*M).  (a) The level of UCP2 mRNA was normalized to 18 s, and UCP2 protein was normalized to *β*-actin. This ratio was set to 100% in the control of BSA. (b) The level of LC3 protein was normalized to *β*-actin, and this ratio was set to 100% in the control of BSA. (c) H4IIE cells were treated with 250**** *μ*M PA for 6 h. Then LC3 puncta formation was observed using an inverted fluorescence microscope. The numbers of LC3 puncta/cell were counted from ≥100 cells. (d) Cells were treated with PA (250**** *μ*M) for 6 h, with or without CQ before being processed; then electron microscope was performed at 40,000x magnification. Data are expressed as the mean ± SD for each experiment. All data presented are representative of three separate experiments with consistent results.

Given that a marked increase in autophagy was observed, we tried to increase intracellular UCP2 expression by UCP2-bearing plasmid transfection to ascertain whether antiautophagy effects could be induced following PA treatment. We first performed WST-1 assays on UCP2 overexpression cells treated with PA at different concentrations and different times. It demonstrated that treatment with PA resulted in a decrease in the levels of cell growth for up to 24 h when compared to BSA-treated cells (Supplementary SFigure 2). UCP2 expression in cells transfected with the UCP2-bearing plasmid was much higher as compared with cells treated with the control vector (Figure 3(a) and Supplementary SFigure 1). First, UCP2 overexpression significantly increased LC3-II levels in cells treated with PA with or without CQ (Figure 3(b)), suggesting that overexpression of UCP2 increased PA-induced autophagy. Furthermore, using inverted fluorescence microscopy (Figure 3(c)), we observed a marked increase of LC3 puncta in PA-treated cells. After all, to investigate autophagosome, electron microscopy analysis was carried out in PA-treated H4IIE cells transfected with the UCP2-bearing plasmid and cells transfected with vector-bearing plasmid acted as a control. As shown in Figure 3(d), more autophagosome structures were observed in UCP2 overexpression cells as compared with control cells by PA treatment under electron microscopy analysis.

**Figure 4:**
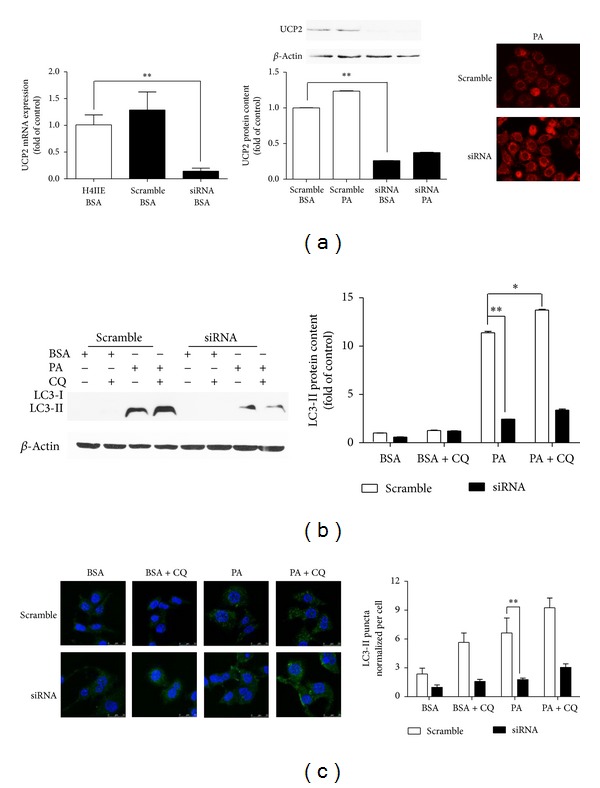
Inhibition of UCP2 decreases PA-mediated autophagy. H4IIE cells were transfected with UCP2-siRNA for 72 h to inhibit the expression of UCP2, followed by 250 *μ*M PA for 6 h, with or without CQ (10 *μ*M). (a) The level of UCP2 mRNA was normalized to 18 s, and UCP2 protein was normalized to *β*-actin. This ratio was set to 100% in the control of BSA. (b) The level of LC3 protein was normalized to *β*-actin, and this ratio was set to 100% in the control of BSA. (c) H4IIE cells were transfected with UCP2 siRNA for 72 h, followed by 250 *μ*M PA for 6 h. Then LC3 puncta formation was observed using an inverted fluorescence microscope. The numbers of LC3 puncta/cell were counted from ≥100 cells. (d) Cells were treated with PA (250 *μ*M) for 6 h, with or without CQ before being processed; then electron microscope was performed at 40,000x magnification. Data are expressed as the mean ± SD for each experiment. All data presented are representative of three separate experiments with consistent results.

To further demonstrate that induction of UCP2 levels is one of the major factors that lead to autophagy following PA treatment, and to confirm the above observations, we next investigated if UCP2 was associated with autophagy in PA-treated cells following UCP2-siRNA transfection. UCP2-siRNA markedly decreased UCP2 mRNA and protein levels in cells after being transfected with UCP2-siRNA for 72 hours (Figure 4(a)). It seems that UCP2-siRNA partially decreased LC3-II levels in cells treated with PA (Figure 4(b)), suggesting that inhibition of UCP2 interferes in PA-induced autophagy. These results indicated that deletion of UCP2 could decrease the effect of PA on LC3 puncta formation, suggesting a positive effect of UCP2 in PA-induced hepatic carcinoma cells autophagy.

### 3.3. PA-Mediated ROS Production Is Partially UCP2 Dependent

**Figure 5:**
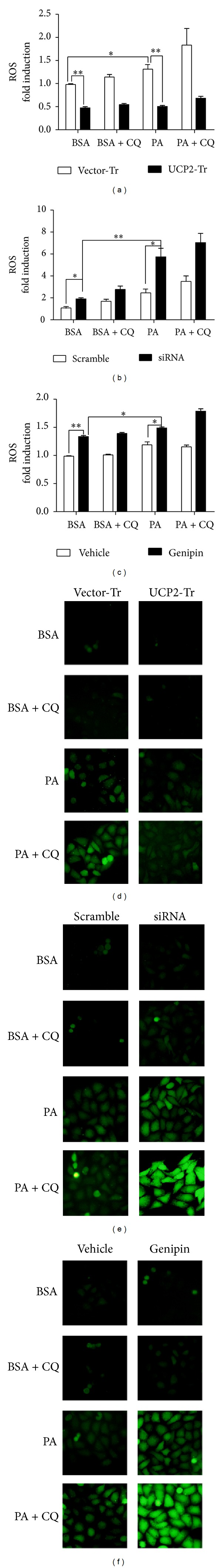
PA-mediated intracellular ROS production is UCP2 mediated. (a) H4IIE cells were transfected with UCP2 plasmid (UCP2-Tr) or control vector (Vector-Tr). Intracellular ROS production induced by 250 *μ*M PA for 6 h was assessed using DCFH-DA and analyzed by flow cytometry. (b) H4IIE cells were transfected with UCP2 siRNA (UCP2-siRNA) or scrambled siRNA (Scramble) for 72 h to inhibit the expression of UCP2 and treated with PA. (c) H4IIE cells were treated with 50 *μ*M genipin for 24 h, followed by PA, with or without CQ (10 *μ*M). (d)–(f) Intracellular ROS was assessed using DCFH-DA staining and microscopy. Data are expressed as the mean ± SD for each experiment. All data presented are representative of three separate experiments with consistent results.

To evaluate the effect of UCP2 on intracellular ROS production, we performed ROS assays on H4IIE cells that were overexpressed UCP2 or inhibited by UCP2 siRNA and genipin. We observed that treatment with PA up to 6 h resulted in a significant increase in ROS production as compared with the BSA-treated control cells using the DCFH-DA assay by flow cytometry (Figures 5(a)–5(c)). This observation was further validated under fluorescence microscopy as shown in Figures 5(d)–5(f). As shown in Figure 5, UCP2 overexpression cells treated with PA resulted in a significant decrease (about 65%) in ROS production as compared with the vector-Tr cells, although overexpressed UCP2 decreases ROS levels in BSA treatments. After inhibiting UCP2 expression, the ROS production of H4IIE cells that were treated with PA increased 60% as compared with the scramble cells. But in BSA treatments the increased ratio is only 40%. Notably, the addition of CQ markedly enhanced ROS production induced by PA in three treatments (overexpression, siRNA, and genipin). However, the addition of CQ to the BSA control cells for up to 6 h did not induce any significant decrease in cell viability, suggesting that CQ alone is not cytotoxic to the H4IIE cells (Figures 5(a)–5(c)).

### 3.4. PA and UCP2 Mediate ATP Synthesis

**Figure 6:**
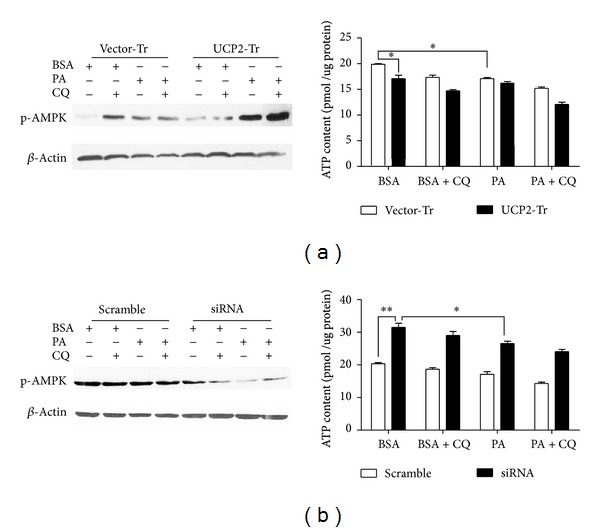
UCP2- and PA-mediated ATP synthesis. (a) H4IIE cells were transfected with UCP2 plasmid (UCP2-Tr) or control vector (Vector-Tr). ATP induced by 250 *μ*M PA for 6 h was assessed using CellTiter-Glo2.0 assay kit, and p-AMPK was normalized to *β*-actin. This ratio was set to 100% in the control of BSA. (b) H4IIE cells were transfected with UCP2 siRNA (UCP2-siRNA) or scrambled siRNA (scramble) for 72 h to inhibit the expression of UCP2 and treated with PA. ATP induced by 250 *μ*M PA for 6 h was assessed using CellTiter-Glo2.0 assay kit, and p-AMPK was normalized to *β*-actin. This ratio was set to 100% in the control of BSA. Data are expressed as the mean ± SD for each experiment. All data presented are representative of three separate experiments with consistent results.

To evaluate the effects of UCP2 on AMPK activation and ATP synthesis, we performed ATP assays.  Figure 6(a) indicated that overexpression of UCP2 could decrease intracellular ATP synthesis. In addition, Figure 6(b) demonstrated that ATP synthesis was increased in UCP2 silencing H4IIE cells.  Figure 6 showed that PA treatment could also decrease intracellular ATP synthesis. UCP2 overexpression or silencing could mediate AMPK activation to some level, but this phenomenon seemed so complicated that needs to be further studied.

### 3.5. UCP2 Has an Antiapoptotic Effect on PA-Induced Apoptosis

**Figure 7:**
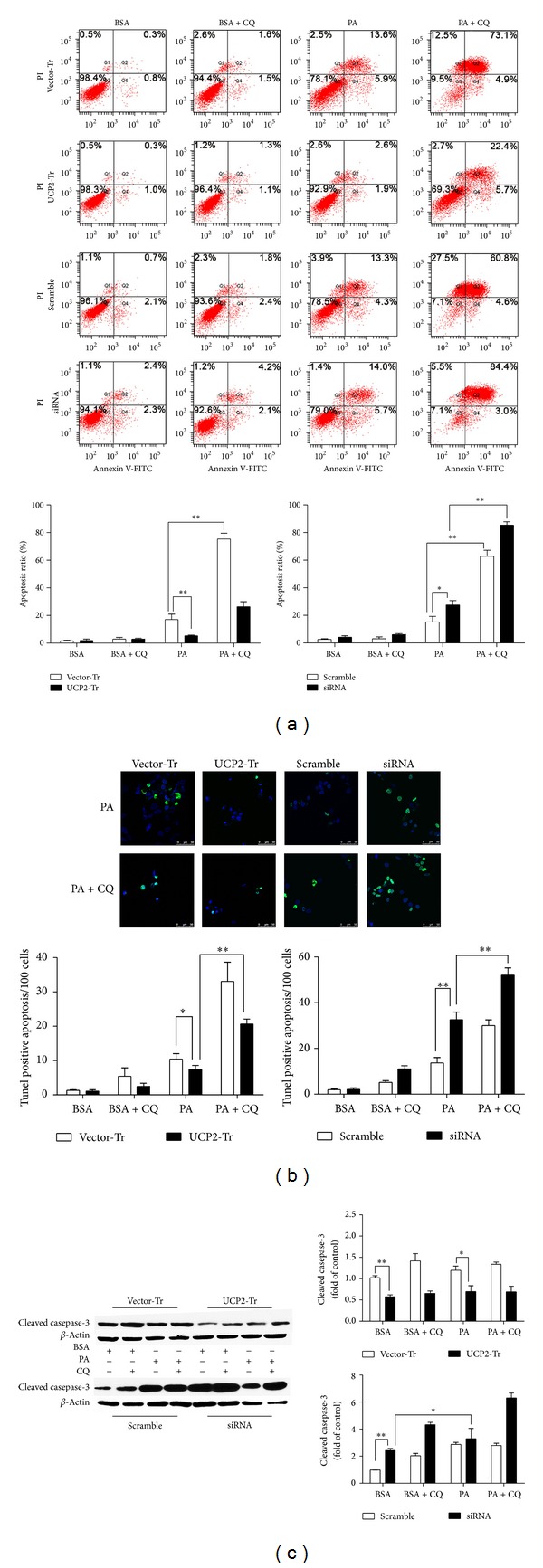
UCP2 has an antiapoptotic effect in PA-induced apoptosis. H4IIE cells were transfected with UCP2-bearing plasmid and UCP2-siRNA and then treated with 250 *μ*M PA for 24 h. (a) Cellular apoptosis induced by 250 *μ*M PA was analyzed by flow cytometry. Data are expressed as the mean ± SD for each experiment. (b) TUNEL-positive cells that were green under fluorescence microscopy were quantified from 100 cells at ×400 magnifications. Data were expressed as the mean ± SD for each experiment. (c) Representative of cleaved caspase-3 and *β*-actin proteins expression by western blot. Data were expressed as the mean ± SD. All data presented are representative of three separate experiments with consistent results.

Recent studies have shown that PA possesses cytotoxic properties [19, 20]. Our latest study has also shown marked cell apoptosis in livers during NASH progression [10]. To further investigate the physiological relevance of UCP2 in the progress of NASH, we treated H4IIE cells with 250 *μ*M PA for up to 24 h. The results showed a significant increase in the number of apoptotic cells when treated with PA compared to BSA-treated cells (Figure 7(a)). It suggested that UCP2 expression may serve as a protective mechanism against lipotoxicity. To assess the relationship between cellular apoptosis and UCP2, we performed the TUNEL assay. PA treatment significantly promoted apoptosis in H4IIE cells, as the numbers of TUNEL-positive cells increased at PA treatments (Figure 7(b)). The same results further confirm that UCP2 plays an antiapoptotic role in PA stimulus. As shown in Figure 7(c), there were higher levels of cleaved caspase-3 in vehicle control cells, which indicated that UCP2 may play an antiapoptotic role and inhibit lipotoxic stress.

As shown in Figure 7, the addition of CQ also enhanced cell death. The addition of CQ enhanced cell death induced by PA. As shown in the BSA control cells, the addition of CQ induced an increase in cell apoptosis. It suggests that autophagy may act as a protective mechanism against apoptosis. This possibility has been reported in a previous study [21], and our data demonstrated a similar mechanism in hepatic carcinoma cells. We observed that treatment with PA resulted in a significant increase in apoptosis as compared with the BSA-treated control cells (Figure 7(a)). This observation was further validated under TUNEL assay as shown in Figure 7(b). As shown in Figure 7, UCP2 overexpression cells treated with PA resulted in a significant decrease (about 200%) in apoptotic cells as compared with the Vector-Tr cells. After inhibiting UCP2 expression, the apoptosis ratio of H4IIE cells that were treated with PA increased 40% as compared with the scramble cells. Notably, the addition of CQ markedly enhanced apoptosis ratio induced by PA in both treatments (overexpression and siRNA). However, the addition of CQ to the BSA control cells did not induce any significant decrease in cell viability, suggesting that CQ alone is not cytotoxic to the H4IIE cells (Figures 5(a)–5(c)).

## 4. Discussion

Lipotoxicity has been thought to be the main contributor to the progression of various diseases associated with excess lipid accumulation in the body, such as obesity and steatohepatitis [22]. The autophagic process has been well documented as a cell survival mechanism and has been implicated in several diseases such as cancer and neurodegenerative diseases [23, 24]. At present, autophagy has been shown to have a role in regulating lipid metabolism. The inhibition of autophagy in cultured hepatocytes and the mouse liver has been shown to increase triglyceride storage in lipid droplets [11], and it is known that PA can regulate autophagic activity in hepatocytes [25]. In this study, we reproved evidence that autophagy can be induced by the saturated fatty acid PA in H4IIe cells. Meanwhile, we showed that autophagy induction by PA is dependent on UCP2 activity. Furthermore, we also present evidence that autophagy plays a prosurvival function to protect against PA-induced lipotoxicity. Our findings are generally consistent with earlier reports that PA is capable of inducing autophagy in pancreatic *β*-cells [26, 27] and embryonic fibroblasts [21]. In a recent study, it has been reported that autophagy can be induced by PA, but not by OA [21]. On the other hand, it is believed that only OA but not PA was capable of inducing autophagy in hepatocytes [28]. One study has also reported that PA prevented fusion of autophagosomes and lysosomes and thus inhibited autophagy [29]. It is believed that cell type, concentration, duration of FFA treatment, and the ratio of conjugated BSA to FFA used could be attributed to these conflicting results.

UCP2 is one of the mitochondrial transporters that are located in the inner mitochondrial membrane and belong to a family of mitochondrial anion carriers, which includes adenine nucleotide transporters. Mild uncoupling of respiration has been reported to diminish mitochondrial reactive oxygen species (ROS) formation [30]. It has been demonstrated that upregulation of UCP2 by AMPK activation attenuates oxidative stress [31]. A recent study also shows that AMPK is an upstream kinase for UCP2 [32]. Our results indicate that UCP2 could decrease intracellular ATP synthesis, and PA stimuli may partially decrease the level of intracellular ATP synthesis. UCP2 is rather an upstream kinase for AMPK than feedback to AMPK activation.

It is well known that UCP2 is an antioxidant mitochondrial protein and that inhibition of UCP2 induces oxidative stress favoring the formation of mitochondrial superoxide ions [33]. Recently, it has been demonstrated that UCP2 is a key redox-sensitive protein [34]. Overexpression of UCP2 decreases cell death following downregulation of ROS production [35]. This aspect of UCP2 function further strengthens the proposition that UCP2 can modulate mitochondrial ROS production and activity. In the present study, we clearly elucidate for the first time the role of UCP2-mediated mitochondrial uncoupling on autophagy regulation in hepatocyte ROS production. The results showed that high UCP2-expressing H4IIE cells have more enhanced adaptive abilities to PA-induced lipotoxicity partly through diminishing ROS production than low UCP2-expressing cells. Because CQ is known to block autophagy by suppressing the lysosomal function, our findings thus indicate that UCP2 may protect against PA-mediated autophagy on ROS production. This aspect of UCP2 function further strengthens the proposition that UCP2 can modulate mitochondrial ROS production and activity [36]. Here, we confirmed that ROS production is mediated by UCP2 after PA-induced hepatocyte lipotoxicity.

Autophagy is a critical intracellular pathway that targets cell constituents to the lysosome for degradation. Recent studies showed that established functions for both macroautophagy and chaperone-mediated autophagy in hepatic lipid metabolism, insulin sensitivity, and cellular injury suggest a number of potential mechanistic roles for autophagy in NASH [15]. Decreased autophagic function in particular may promote the initial development of hepatic steatosis and progression of steatosis to liver injury [15]. In the present study, we observed that UCP2 overexpression also significantly increased autophagy in PA-treated cells, while inhibition of UCP2 resulted in a decrease in PA-induced autophagy. Furthermore, with loss of autophagic function, decreased lipid storage occurred, and an increased rate of fatty acid *β*-oxidation was observed [12]. We suspect that PA-induced autophagy occurred partly through increased UCP2 upregulation.

In this study, we have shown that UCP2 is associated with apoptosis induced by fatty acids* in vitro*. Our previous study has shown that PA was able to induce liver damage that resembles NAFLD in humans and was characterized by increasing caspase-3 activity and prominent apoptosis [10]. To further confirm these results, we altered UCP2 expression levels in H4IIE hepatoma cells by transfection with either an UCP2 mRNA interference (siRNA) plasmid or a UCP2-overexpressing plasmid. UCP2 overexpression caused significantly decreased apoptosis rates and caspase-3 activity in the PA-treated cells while UCP2 siRNA resulted in an increase in apoptosis rates and caspase-3 activity. Overall, we have demonstrated that UCP2 protects hepatic carcinoma cells from PA-induced apoptosis* in vitro* by increasing hepatocyte autophagy. We predict that the antiapoptotic effect of UCP2 most likely relates to its preventative role in its inductive effect of hepatoma autophagy.

## 5. Conclusion

Our present study shows that UCP2 was upregulated and that hepatocellular autophagy was increased during PA treatment. Increasing UCP2 expression in hepatoma cells may contribute to cell autophagy. Hepatic autophagy play a protective role in hepatocyte lipoapoptosis. The results provide evidence that UCP2 is a proliferative factor that also has an antiapoptotic role during PA-induced liver injury. The current data obtained from our experiments may provide useful information regarding potential molecular targets for NASH prevention and treatment.

## Supplementary Material

Supplement FIGURE 1: LC3-II level increased by PA in UCP2-Tr cells. UCP2 over-expression H4IIE cells were treated with PA (250 *μ*M) conjugated to fatty acid-free BSA at different time points. H4IIE Cells treated with BSA acted as a control. After the treatment, cell lysates were collected and subjected to western blotting. Data are expressed as the mean ±SD for each experiment. All data presented are representative of three separate experiments with consistent results.
Supplement FIGURE 2: The effects of PA on UCP2 over-expression cell . UCP2 over-expression H4IIE cells were treated with PA (6 h) conjugated to fatty acid-free BSA at different concentrations, or H4IIE cells were treated with PA (250 *μ*M) conjugated to fatty acid-free BSA at different time points. H4IIE Cells treated with BSA acted as a control. After treatments, cells were stained and subjected to the WST-1 assay. Data are expressed as the mean ±SD for each experiment. All data presented are representative of three separate experiments with consistent results.
Supplement FIGURE 3: PA induces UCP2 expression in Brl cells. Brl cells were treated with PA (6 h) conjugated to fatty acid-free BSA or H4IIE cells treated with BSA. Brl Cells treated with BSA acted as a control. After treatments, cells were stained and subjected to the WST-1 assay. Data are expressed as the mean ±SD for each experiment. All data presented are representative of three separate experiments with consistent results.
Supplement FIGURE 4: OA induces autophagy in H4IIE cells. H4IIE cells were treated with OA (250 *μ*M) conjugated to fatty acid-free BSA for (2, 4, 6, 8, 12, and 24 h) as indicated. Cells treated with BSA acted as a control. After the treatment, cell lysates were collected and subjected to western blotting. Data are expressed as the mean ±SD for each experiment. All data presented are representative of three separate experiments with consistent results.
Supplement FIGURE 5: The effects of CQ on H4IIE cell. H4IIE cells were treated with CQ (24 h) conjugated to fatty acid-free BSA for (2.5, 5, 10, 20, and 50 *μ*M) as indicated. Cells treated with BSA acted as a control. After treatments, cells were stained and subjected to the WST-1 assay. Data are expressed as the mean ±SD for each experiment. All data presented are representative of three separate experiments with consistent results.

## Abbreviations

ROS: Reactive oxygen species
UCP2: Uncoupling protein 2
NAFLD: Nonalcoholic fatty liver disease
NASH: Nonalcoholic steatohepatitis
FFA: Free fatty acid
PA: Palmitic acid
CQ: Chloroquine diphosphate.
