# Supplementary material for: Uncoupling Protein 2 Regulates Palmitic Acid-Induced Hepatoma Cell Autophagy
Source: Biomed Res Int. 2014 Aug 4;2014:810401. doi: 10.1155/2014/810401 (PMC4143590; doi:10.1155/2014/810401)
Supplement: Supplementary file 1 — Supplement FIGURE 1: LC3-II level increased by PA in UCP2-Tr cells. UCP2 over-expression H4IIE cells were treated with PA (250 μM) conjugated to fatty acid-free BSA at different time points. H4IIE Cells treated with BSA acted as a control. After the treatment, cell lysates were collected and subjected to western blotting. Data are expressed as the mean ±SD for each experiment. All data presented are representative of three separate experiments with consistent results. Supplement FIGURE 2: The effects of PA on UCP2 over-expression cell . UCP2 over-expression H4IIE cells were treated with PA (6 h) conjugated to fatty acid-free BSA at different concentrations, or H4IIE cells were treated with PA (250 μM) conjugated to fatty acid-free BSA at different time points. H4IIE Cells treated with BSA acted as a control. After treatments, cells were stained and subjected to the WST-1 assay. Data are expressed as the mean ±SD for each experiment. All data presented are representative of three separate experiments with consistent results. Supplement FIGURE 3: PA induces UCP2 expression in Brl cells. Brl cells were treated with PA (6 h) conjugated to fatty acid-free BSA or H4IIE cells treated with BSA. Brl Cells treated with BSA acted as a control. After treatments, cells were stained and subjected to the WST-1 assay. Data are expressed as the mean ±SD for each experiment. All data presented are representative of three separate experiments with consistent results. Supplement FIGURE 4: OA induces autophagy in H4IIE cells. H4IIE cells were treated with OA (250 μM) conjugated to fatty acid-free BSA for (2, 4, 6, 8, 12, and 24 h) as indicated. Cells treated with BSA acted as a control. After the treatment, cell lysates were collected and subjected to western blotting. Data are expressed as the mean ±SD for each experiment. All data presented are representative of three separate experiments with consistent results. Supplement FIGURE 5: The effects of CQ on H4IIE cell. H4IIE cells were t [file 810401.f1.doc]

**SFig 1**

**
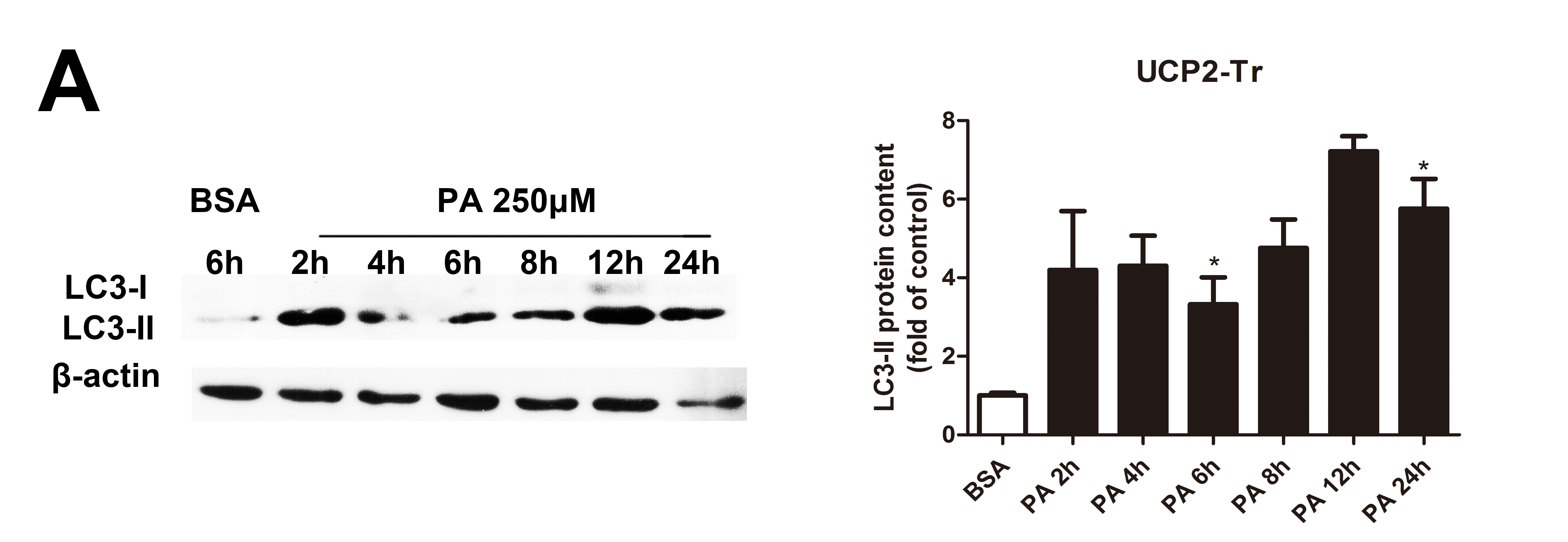
**

Supplement FIGURE 1. LC3-II level increased by PA in UCP2-Tr cells. UCP2 over-expression H4IIE cells were treated with PA (250 µM) conjugated to fatty acid-free BSA at different time points. H4IIE Cells treated with BSA acted as a control. After the treatment, cell lysates were collected and subjected to western blotting. Data are expressed as the mean ±SD for each experiment. All data presented are representative of three separate experiments with consistent results.

**SFig 2**

**
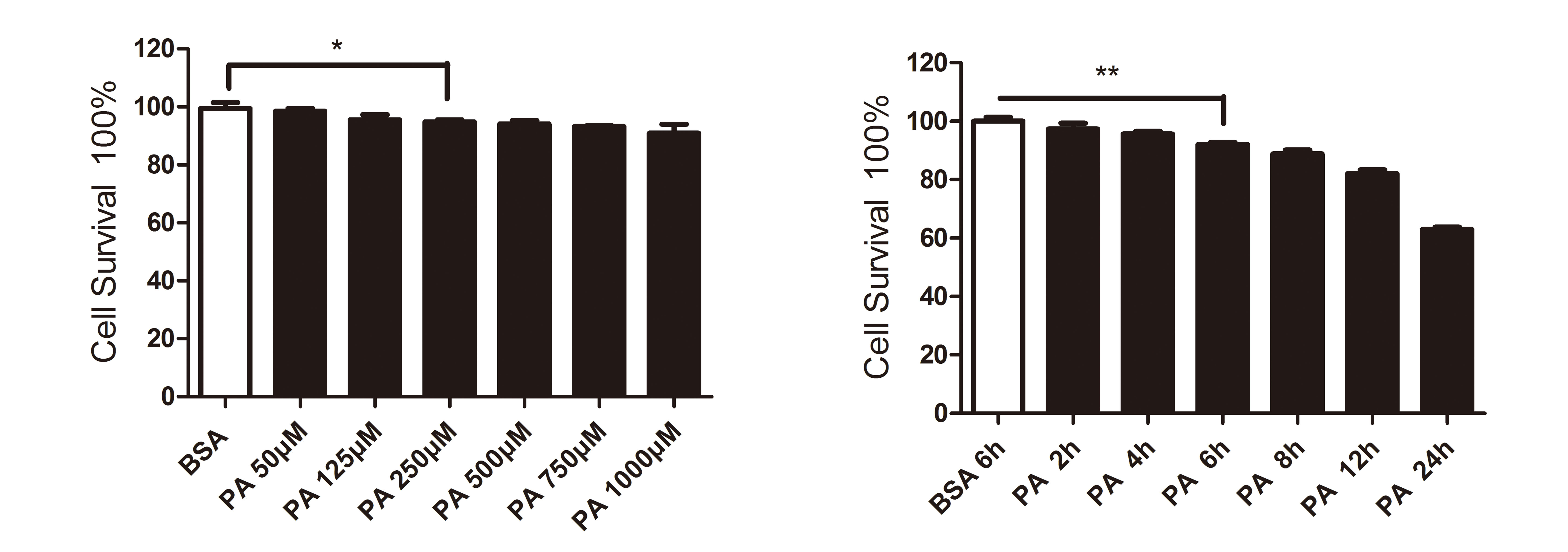
**

Supplement FIGURE 2. PA induces UCP2-Tr cell death. UCP2 over-expression H4IIE cells were treated with PA (6 h) conjugated to fatty acid-free BSA at different concentrations, or H4IIE cells were treated with PA (250 µM) conjugated to fatty acid-free BSA at different time points. H4IIE Cells treated with BSA acted as a control. After treatments, cells were stained and subjected to the WST-1 assay. Data are expressed as the mean ±SD for each experiment. All data presented are representative of three separate experiments with consistent results.

**SFig 3**

**
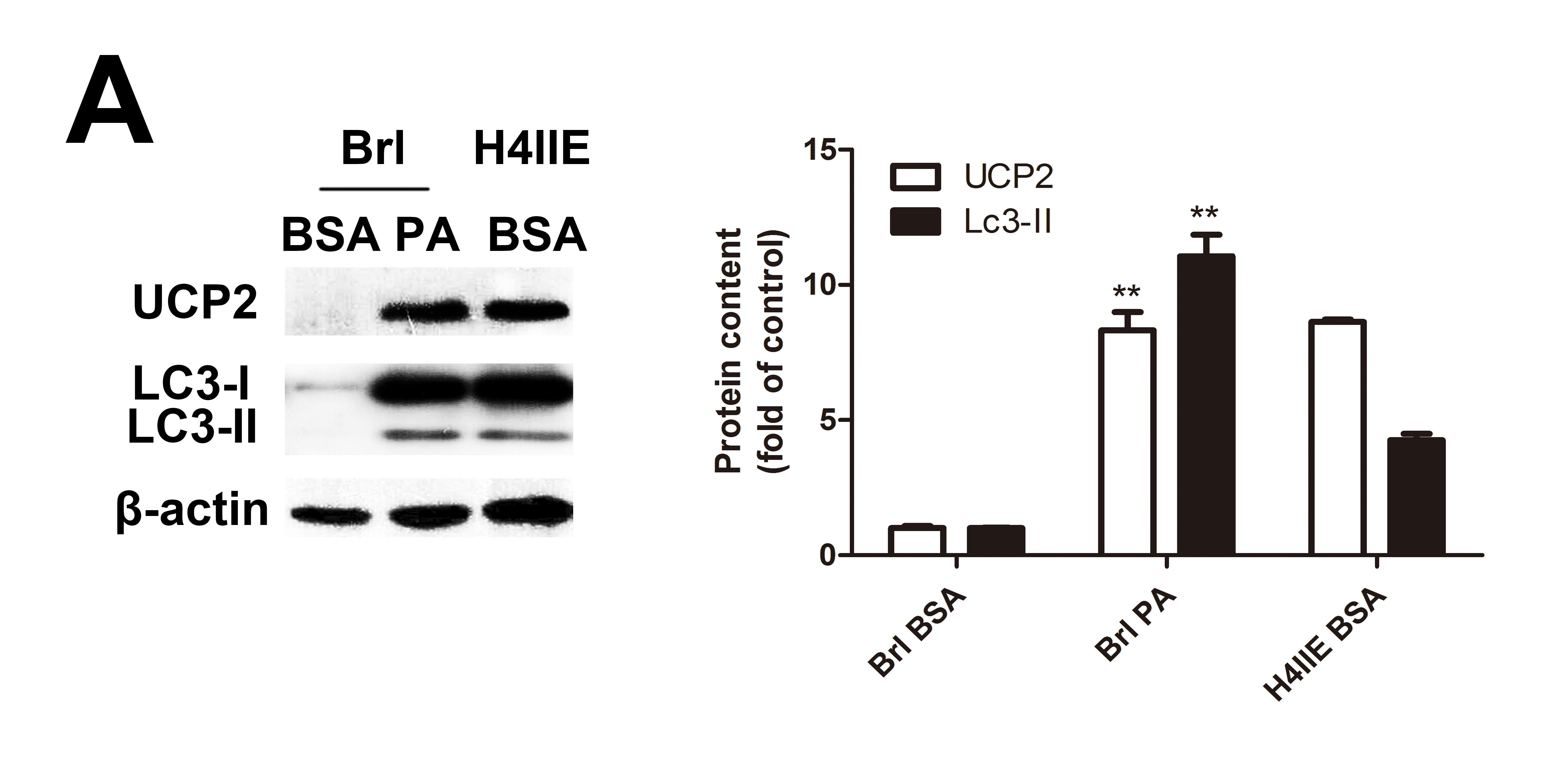
**

Supplement FIGURE 3. PA induces UCP2 expression in Brl cells. Brl cells were treated with PA (6 h) conjugated to fatty acid-free BSA or H4IIE cells treated with BSA. Brl Cells treated with BSA acted as a control. After treatments, cells were stained and subjected to the WST-1 assay. Data are expressed as the mean ±SD for each experiment. All data presented are representative of three separate experiments with consistent results.

**SFig 4**

**
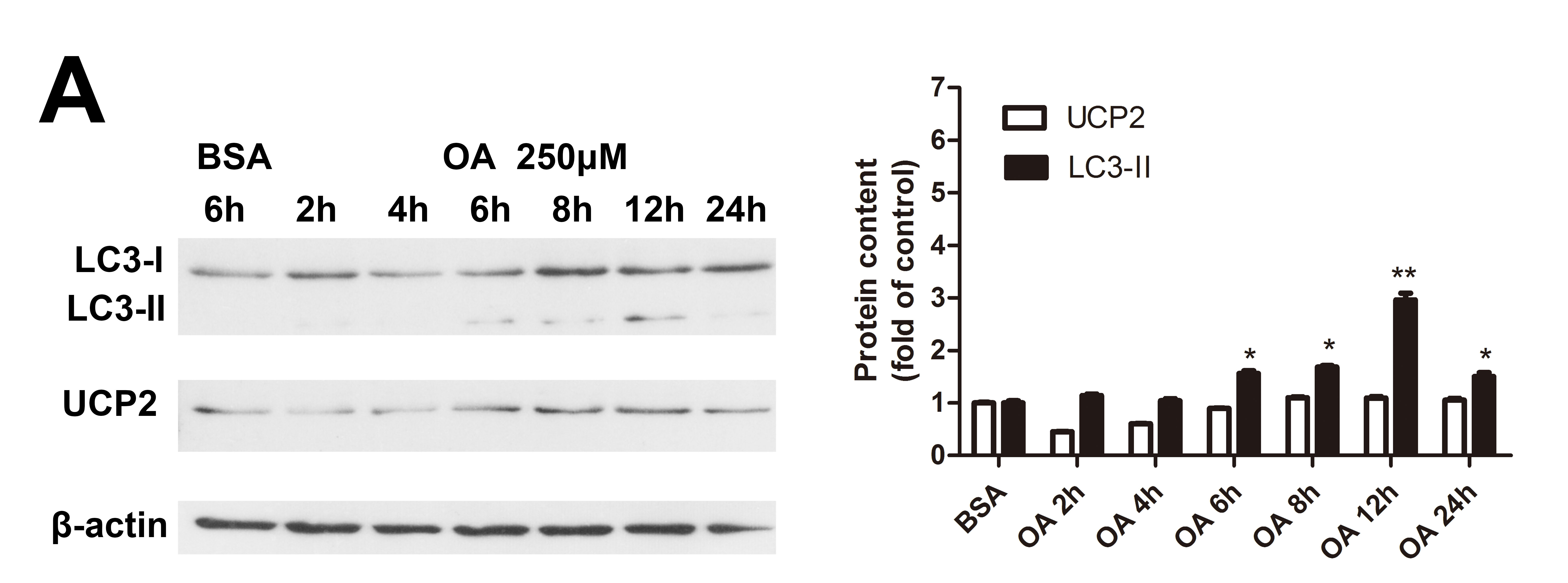
**

Supplement FIGURE 4. OA induces autophagy in H4IIE cells. H4IIE cells were treated with OA (250 µM) conjugated to fatty acid-free BSA for (2, 4, 6, 8, 12, and 24 h) as indicated. Cells treated with BSA acted as a control. After the treatment, cell lysates were collected and subjected to western blotting. Data are expressed as the mean ±SD for each experiment. All data presented are representative of three separate experiments with consistent results.

**SFig.5**

**
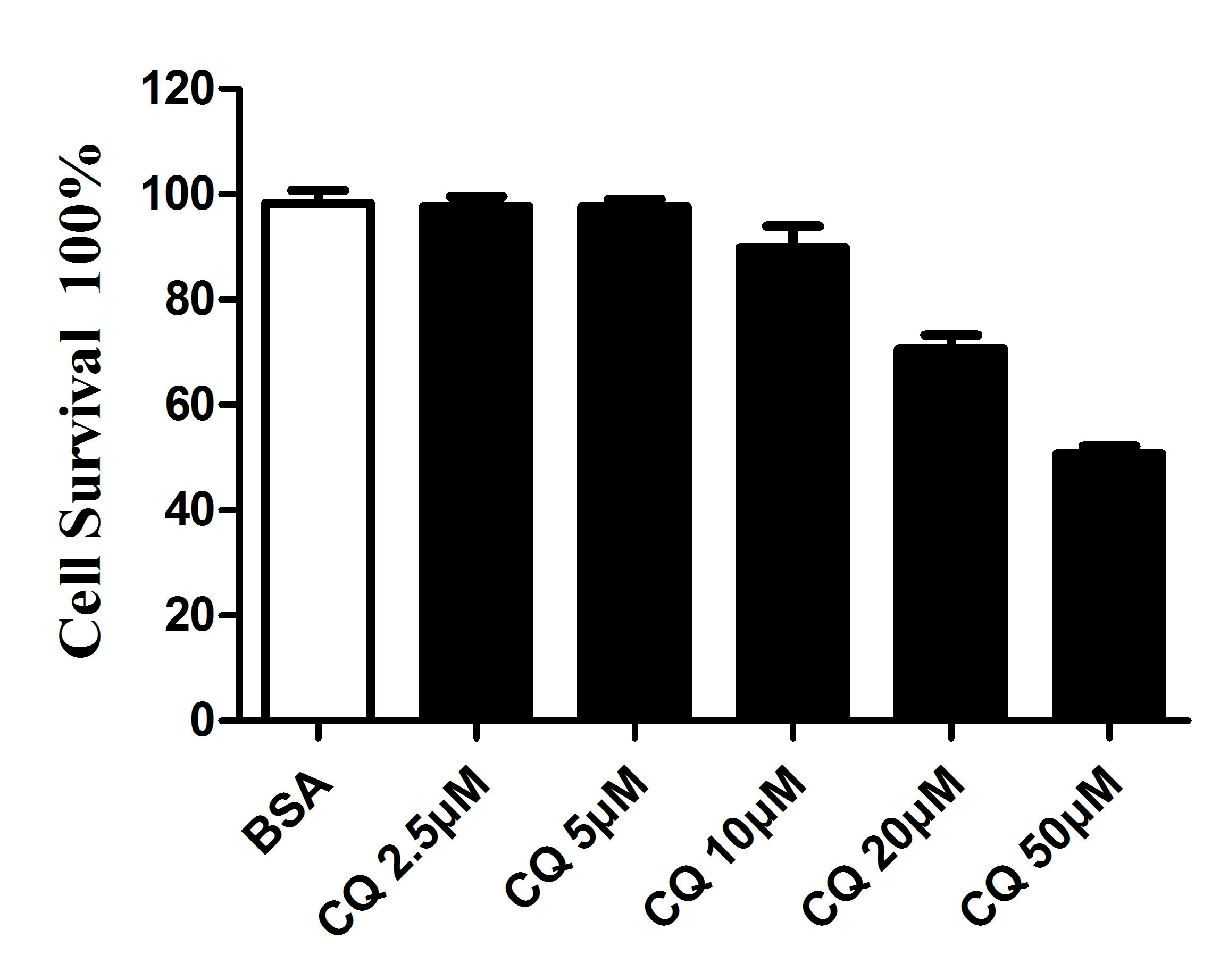
**

Supplement FIGURE 5. CQ induces H4IIE cell death. H4IIE cells were treated with CQ (24 h) conjugated to fatty acid-free BSA for (2.5, 5, 10, 20, and 50 µM) as indicated. Cells treated with BSA acted as a control. After treatments, cells were stained and subjected to the WST-1 assay. Data are expressed as the mean ±SD for each experiment. All data presented are representative of three separate experiments with consistent results.
